# Draft genome sequence of *Pseudomonas* sp. GOM6, a lipolytic strain isolated from seawater of the Gulf of Mexico

**DOI:** 10.1128/MRA.00348-23

**Published:** 2023-07-20

**Authors:** Jorge Rojas-Vargas, Luis Felipe Muriel-Millán, Liliana Pardo-López

**Affiliations:** 1 Instituto de Biotecnología, UNAM, Cuernavaca, Morelos, Mexico; Montana State University, Bozeman, Montana, USA

**Keywords:** Gulf of Mexico, lipolytic activity, bacteria

## Abstract

We report here the draft genome sequence of a marine *Pseudomonas* sp. novel species with lipase activity isolated from a deep-sea water sample of the Gulf of Mexico. The genome consists of 4.3 Mbp in 48 contigs.

## ANNOUNCEMENT

The *Pseudomonas* genus comprises 526 recognized species and 23 subspecies (https://lpsn.dsmz.de/search?word=pseudomonas). Some have been discovered in marine environments, such as strains of *P. aeruginosa, P. putida*, and *P. chengduensis* (1 - 3). Examining the genomes of these environmental bacteria increases our understanding of microorganism biology and their potential applications in biotechnology. Marine bacterial enzymes such as proteases, oxygenases, lipases, and esterases are of interest for their versatile catalytic activities (4).

We introduce the draft genome of *Pseudomonas* sp. GOM6, a strain isolated from a seawater sample from the Gulf of Mexico’s Perdido Fold Belt area (25.83 latitude, −95.50 longitude) in September 2015. The sample was obtained with a conductivity temperature depth (CTD) rosette sampler at a depth of 1,000 m, and 1 mL of the sample was inoculated into 30 mL of PY medium supplemented with 1% Bacab alfa crude oil (5). The culture was stored at 4–8°C for 8 weeks until its processing. Lipolytic bacterial strains were isolated by spreading a diluted culture (100 µL aliquot of a 10^−4^ dilution) on modified Lysogeny Broth (LB) plates (0.5% peptone, 0.3% yeast extract, 1% tributyrin, 1% arabic gum, and 1.3% bacto agar) (6). After incubation at 30°C (48 h), colonies displaying hydrolytic halos were selected and grown on modified LB plates with 1% tricaprylin for lipase activity detection (6).

*Pseudomonas* sp. GOM6 was grown overnight on LB broth at 30°C, 180 RPM, followed by genomic DNA extraction using the Quick-gDNA miniprep kit (Zymo Research, Irvine, CA, USA). Illumina MiSeq platform was used for sequencing at the Massive Sequencing Unit of the Institute of Biotechnology (IBt-UNAM, Cuernavaca, Mexico). Illumina sequencing library was prepared using the Nextera library kit (Illumina, Inc.), where DNA was fragmented enzymatically. The sequencing resulted in 2,154,161 paired-end reads with a read length of 75 bp and a median sequence quality (Phred score) of 35. The read quality was examined using the FastQC software v0.11.9 (7), and quality was filtered and trimmed using Trimmomatic v0.39 (8).

Genome *de novo* assembly was made with SPAdes assembler v3.13.0 (9). Quality analysis was performed using QUAST v5.0.2 (10) and the completeness and contamination with CheckM v1.2.2 (11). The genome contains 4,305,958 bp in 48 contigs with an N50 value of 246,512 bp and a GC content of 62.81%. Completeness of 98.86% and contamination of 1.08% were obtained, with a 49× sequencing depth. A plasmid was detected *in silico* using PlasmidSPAdes (12), with 252,791 bp and coverage 26×, corresponding to the contig 5 of the assembly.

**Fig 1:**
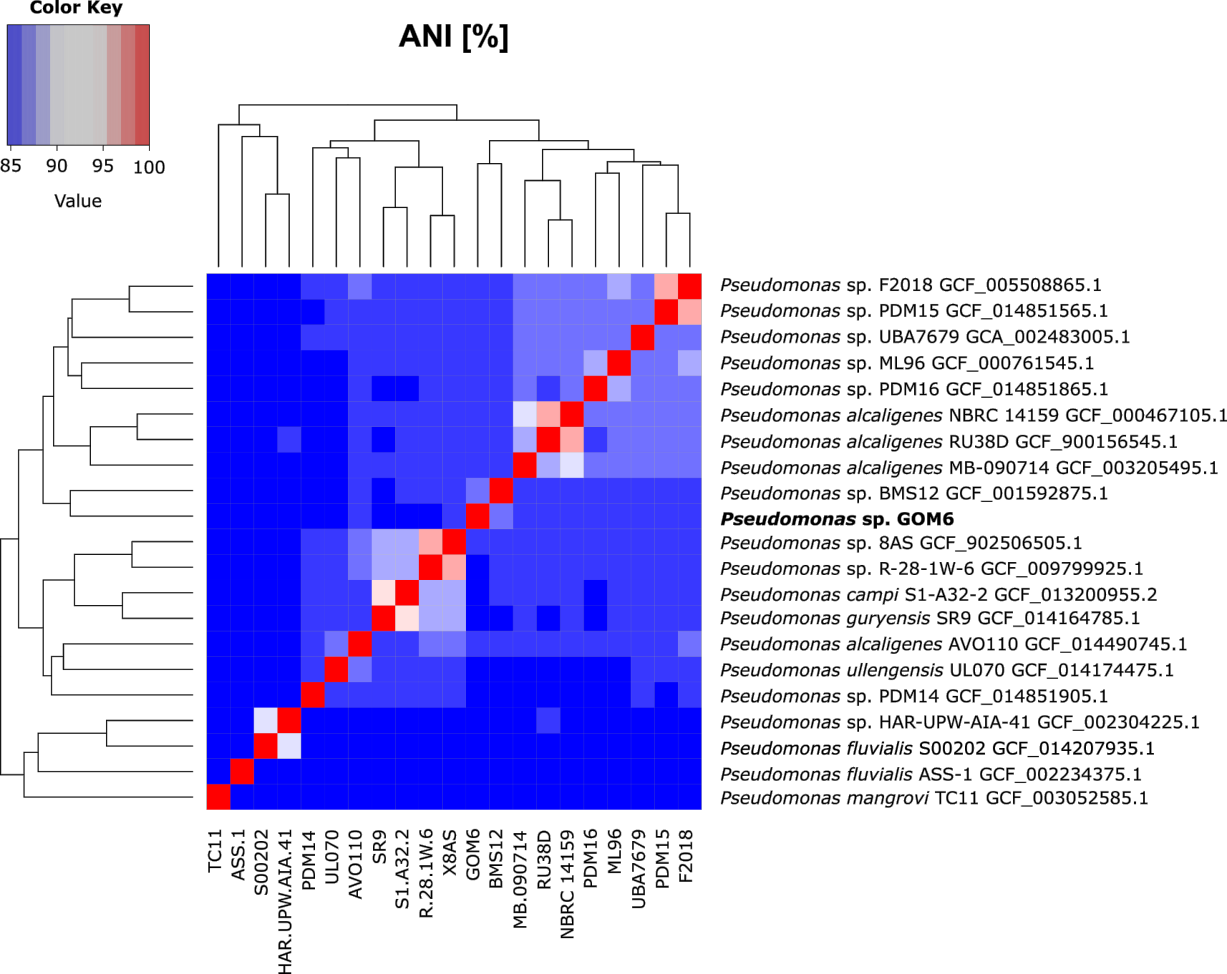
Heatmap of ANI values calculated with PyANI. Higher values correspond with greater genetic similarity between strains (red). The GOM6 assembly has 67.92% alignment with the BMS12 genome and less than 60% with the other genomes.

The average nucleotide identity (ANI) values were calculated using PyANI v0.2.12 (https://github.com/widdowquinn/pyani) against the 20 closest genomes determined by GTDBtk v2.1.0 (13) (Fig. 1). The assemblies were retrieved from the NCBI portal (https://www.ncbi.nlm.nih.gov/assembly consulted on 18 March 2023). The highest ANI value was against the genome of *Pseudomonas* sp. BMS12 (14) (ANI of 88.19%) suggests that GOM6 is a novel *Pseudomonas* species.

Gene prediction and functional annotation were performed with the NCBI Prokaryotic Genome Annotation Pipeline (15) and reported 4,111 genes, 4,061 protein-coding genes, and 50 RNAs. Default parameters were used for all software unless otherwise specified.

## Data Availability

The whole-genome sequence data and raw sequences are available at NCBI under the accession number ASM2953748v1, Bioproject accession number PRJNA948904, and Sequence Read Archive (SRA) accession numbers SRR24154338.
